# Preoperative joint line convergence angle correction is a key factor in optimising accuracy in varus knee correction osteotomy

**DOI:** 10.1007/s00167-022-07092-2

**Published:** 2022-08-22

**Authors:** P. Behrendt, R. Akoto, I. Bartels, G. Thürig, H. Fahlbusch, A. Korthaus, D. Dalos, M. Hoffmann, K.-H. Frosch, M. Krause

**Affiliations:** 1 Department of Orthopedic and Trauma Surgery, Asklepios St. Georg, Hamburg, Germany; 2 Department of Trauma and Orthopaedic Surgery, University Medical Center Hamburg-Eppendorf, Hamburg, Germany; 3 Department of Anatomy, Christian-Albrechts-University, Kiel, Germany; 4 Department of Trauma and Orthopaedic Surgery, Sports Traumatology, BG Hospital Hamburg, Hamburg, Germany; 5 Department of Traumatology, HFR Fribourg Hospital Cantonal, Fribourg, Switzerland; 6 UKE Athleticum-Center for Athletic Medicine, University Medical Center Hamburg-Eppendorf, 20246 Hamburg, Germany; 7 Cologne Merheim Medical Center, University of Witten/Herdecke, Cologne, Germany

**Keywords:** Osteotomy, Varus, HTO, JLCA, Accuracy, Planning, Overcorrection, MPTA

## Abstract

**Purpose:**

This study aimed to identify and prevent preoperative factors that can be influenced in preoperative planning to reduce postoperative malcorrections.

**Methods:**

The method used in this study was a retrospective two-centre analysis of 78 pre and postoperative fully weight-bearing radiographs of patients who underwent valgus osteotomy correction due to symptomatic medial compartment osteoarthritis. A computer software (TraumaCad^®^) was used to aim for an intersection point of the mechanical tibiofemoral axis (mTFA) with the tibia plateau at 55–60% (medial = 0%, lateral = 100%). Postoperative divergence ± 5% of this point was defined as over- and undercorrection. Preoperative joint geometry factors were correlated with postoperative malcorrection. Planning was conducted using the established method described by Miniaci (Group A) and with additional correction of the joint line convergence angle (JLCA) using the formula JLCA-2/2 (Group B). Additionally, in a small clinical case series, planning was conducted with JLCA correction. Statistical analysis was performed using (multiple) linear regression analysis and analysis of variance (ANOVA) with *p* < 0.05 considered significant.

**Results:**

In 78 analysed cases, postoperative malcorrection was detected in 37.2% (5.1% undercorrection, 32.1% overcorrection). Linear regression analysis revealed preoperative body mass index (BMI, *p* = 0.04), JLCA (*p* = 0.0001), and osteotomy level divergence (*p* = 0.0005) as factors correlated with overcorrection. In a multiple regression analysis, JLCA and osteotomy level divergence remained significant factors. Preoperative JLCA correction reduced the planned osteotomy gap (A 9.7 ± 2.8 mm vs B 8.3 ± 2.4 mm; *p* > 0.05) and postoperative medial proximal tibial angle (MPTA: A 94.3 ± 2.1° vs B 92.3 ± 1.5°; *p* < .05) in patients with preoperative JLCA ≥ 4°. The results were validated using a virtual postoperative correction of cases with overcorrection. A case series (*n* = 8) with a preoperative JLCA > 4 revealed a postoperative accuracy using the JLCA correction of 3.4 ± 1.9%.

**Conclusion:**

Preoperative JLCA ≥ 4° and tibial osteotomy level divergence were identified as risk factors for postoperative overcorrection. Preoperative JLCA correction using the formula JLCA-2/2 is proposed to better control ideal postoperative correction and reduce MPTA. The intraoperatively realised osteotomy level should be precisely in accordance with preoperative planning.

**Level of evidence:**

III, cross-sectional study.

## Introduction

Limited accuracy in osteotomy correction of varus malalignment in the knee is an unsolved issue of tremendous importance since bony corrections are applied with increasing frequency for many indications, such as unicompartmental osteoarthritis (OA), instabilities, and corrections for aesthetical reasons [8, 23, 37].

Several factors have already been identified to optimise the predictive power of the preoperatively desired correction target, including preoperative planning, according to Miniaci and others [1, 14, 17, 41]. Navigated osteotomy guidance has been investigated, as it seems to be a promising technique, but it has not yet been proven to improve osteotomy accuracy [38]. Nevertheless, there remains a considerable lack of accuracy in knee osteotomies concerning the postoperatively achieved correction target [37]. Depending on the definition of postoperative malcorrection, the rate of undesired overcorrection has been reported to be up to 40% [10, 40]. Overcorrection, as well as certain aspects of postoperative joint geometry like a medial proximal tibia angle (MPTA) > 92° and an oblique joint line (JLO), may affect the clinical outcome and complicate later prosthesis implantation [15, 31, 40].

In this regard, an essential factor not finally considered in osteotomy planning is the proportion of intraarticular varus, its associated soft tissue balancing, and how to deal with it in preoperative planning. The joint line convergence angle (JLCA) is a known surrogate measure to estimate the amount of intraarticular malalignment [7] and has been associated with postoperative malcorrection [16, 32, 33]. Many efforts have been undertaken to estimate perioperative JLCA change [7, 27], but all require additional radiographs and have certain limitations. Micicoi et al. introduced a simple equation JLCA-2/2 that estimates the average JLCA change from pre- to postoperatively; however, its clinical application in planning has not been validated [25].

The purpose of this study was to identify preoperative factors associated with postoperative malcorrection and validate preoperative planning with or without JLCA-2/2-correction to improve overall osteotomy accuracy. The authors hypothesise that an increased preoperative JLCA is associated with postoperative malcorrections using the conventional Miniaci planning method. We further hypothesise that preoperative JLCA should be considered in preoperative planning to improve overall osteotomy accuracy.

## Materials and methods

Patients who underwent varus malalignment correction between 2016 and 2021 at the authors’ institution were included in this two-centre study after approval by the Ethics Committee of the University of Hamburg. Indications for valgus osteotomy were unicompartmental OA and focal cartilage lesions with concomitant cartilage repair procedures. Exclusion criteria were skeletal immaturity, post-traumatic deformities, previous surgery with the affection of the lower extremity alignment, and previous hip, knee, or ankle replacement. No corrections due to ligament injuries or intraoperative hinge fractures were included because they could affect the JLCA independently from any bony deformity. In addition, valgus osteotomies performed at the distal femur (distal femur osteotomy) and double-level osteotomies were excluded.

Particular attention was paid to the accuracy of the pre-and postoperative radiographs, as malrotation radiographs can significantly alter the measurements [3]. In properly aligned long-leg weight-bearing X-rays, the patella should be centred between the femur condyles and forward-facing. The femur condyles should be symmetrically aligned and parallel to the frontal plane. The fibulotalar joint should not be fully visible, and the lateral tibial plateau should partially cover the head. Patients with inaccurate (malrotated) radiographs and knee extension deficits were excluded.

Deformity analysis and osteotomy simulation were performed using computer-based planning software (TraumaCAD™, Petach-Tikva, Israel). Measurements were conducted by a senior orthopaedic fellow and an orthopaedic consultant.

**Fig. 1:**
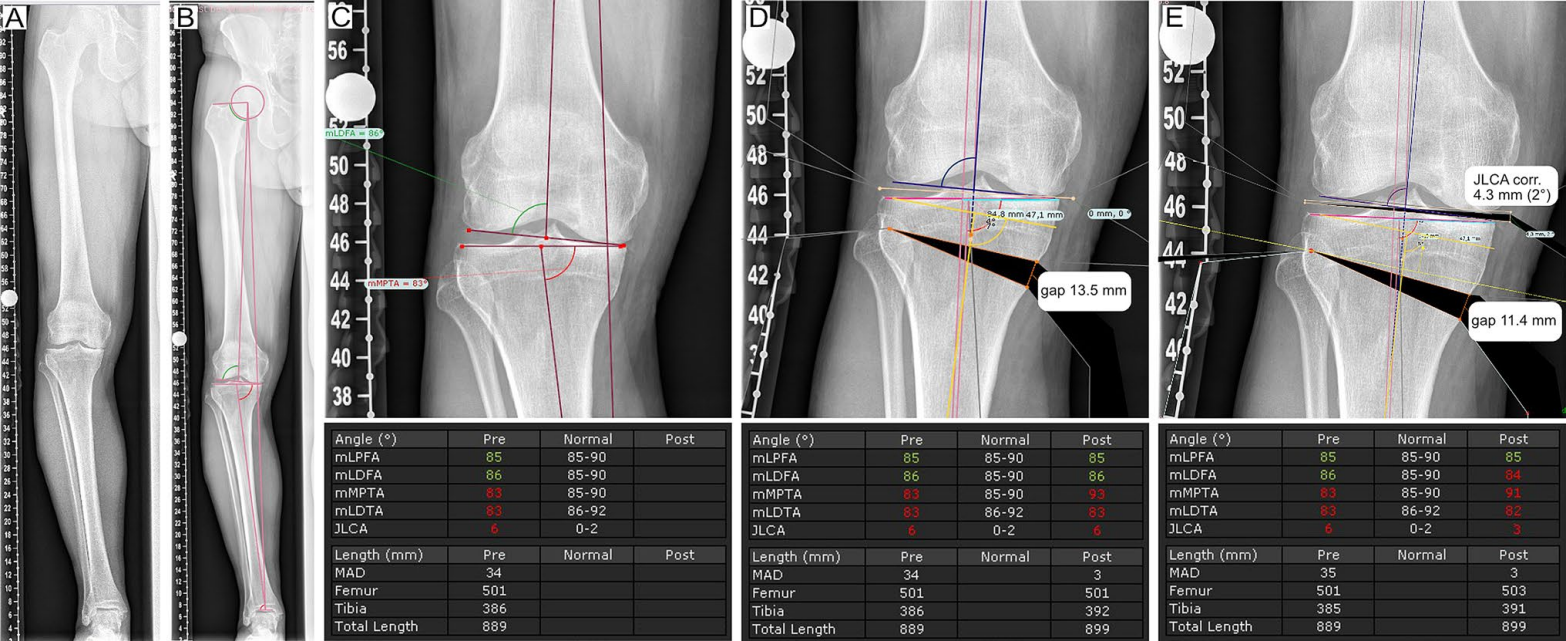
Preoperative osteotomy planning. **A**–**C** Based on long-leg weight-bearing X-rays, a deformity analysis was conducted preoperatively. **D** After defining the CORA, a software-guided osteotomy simulation (TraumaCAD™) was conducted based on the technique described by Miniaci. **E** Modified planning was performed with a previous JLCA correction using the JLCA-2/2 formula and subsequent OW high tibia osteotomy. The influence of the simulated osteotomy is directly visualised by the change of the geometrical joint parameters “Pre” (operative) and “Post” (operative)

For each patient, pre-and postoperative radiographs were imported to TraumaCad™, calibrated, and analysed for the following parameters as described by Paley et al. (Fig. 1A–C):

- Mechanical femorotibial angle (mTFA).
- The tibial intersection of the weight-bearing line (WBL ratio; expressed as % of the medial (0%)-to-lateral (100%) width of the tibial plateau).
- Mechanical medial proximal tibia angle (mMPTA).
- Mechanical lateral distal femur angle (mLDFA).
- Joint line obliquity (JLO).
- JLCA.
- Mechanical axis deviation (MAD)
- Hip knee angle (HKA)

Based on the centre of rotation and angulation (CORA) principle, the bony correction was simulated using the technique described by Miniaci et al. and conducted as open-wedge (OW) high tibial osteotomy (HTO). Depending on the osteotomy indication, the mTFA was planned to be a 55–60% WBL ratio (Fig. 1D).

**Fig. 2:**
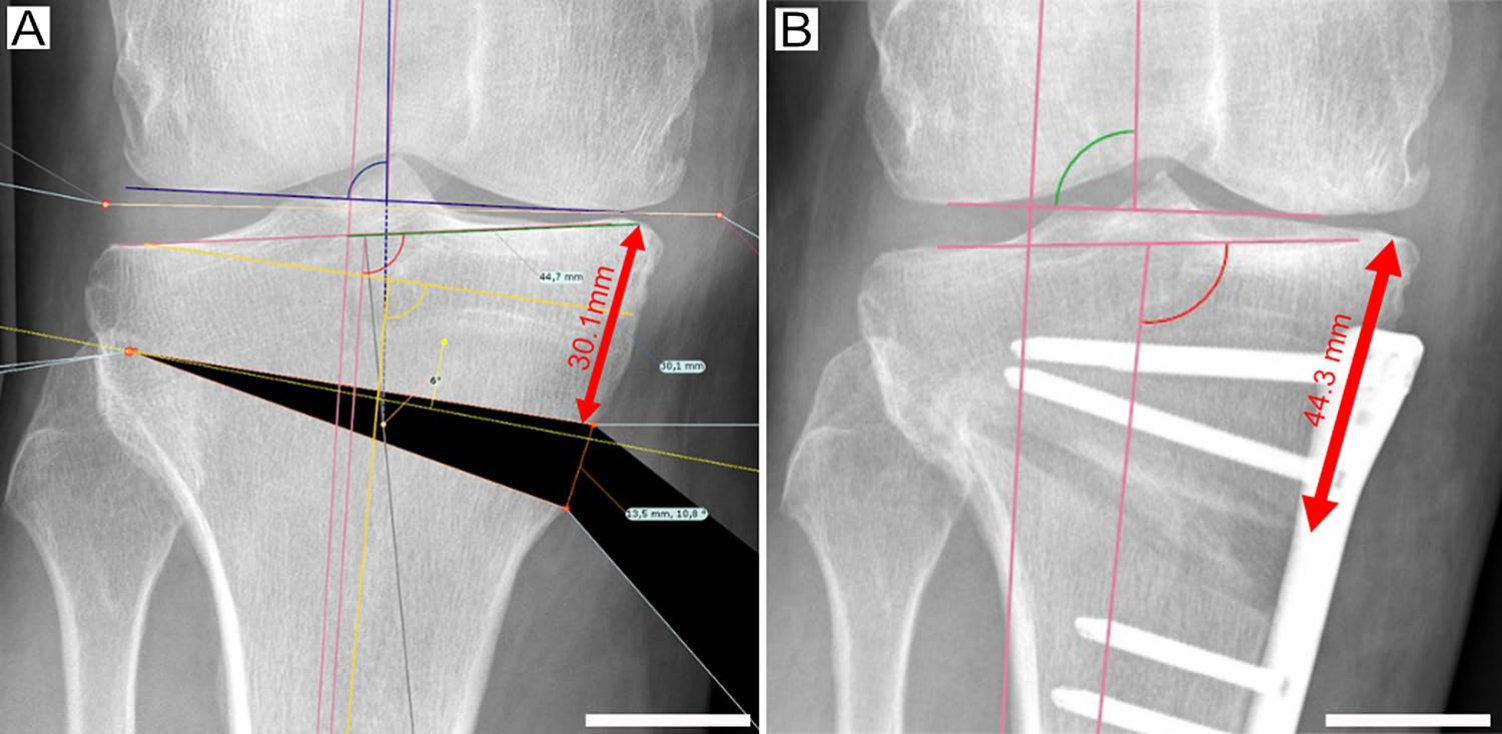
Osteotomy level deviance. Illustration of the different osteotomy levels in **A** preoperative osteotomy simulation and **B** intraoperative realisation of the same patient. The osteotomy level was measured as the distance from the tibial plateau to the medial osteotomy gap. Scale bar 25 mm

The osteotomy level (Fig. 2) was defined as the cortical distance from the tibial plateau to the medial osteotomy. It was standardised to 35 mm, corresponding to the internal fixateur used in this study (4.5 LOQTEQ plate system, AAP Implantate AG, Germany) and prevention of plate positioning close to the joint line, which can cause postoperative pain. A different osteotomy level of the preoperative planning and intraoperative realisation measured at the postoperative radiographs was defined as “divergence of the osteotomy level”.

Modified preoperative planning was conducted by adding a previous step for JLCA correction (Fig. 1E). Therefore, an intraarticular osteotomy was simulated and corrected by an intraarticular correction angle calculated using the JLCA-2/2 formula described by Micicoi et al. [25]. Afterwards, the osteotomy was conducted as described by Miniaci et al. [27]. For example, in a case with a preoperative JLCA of 6°, an additional intraarticular osteotomy of 6°–2°/2 = 2° was conducted prior to the OW HTO of the proximal tibia. No JLCA correction was performed for preoperative JLCA values smaller than or equal to 2° (2°–2°/2 = 0°). For the surgical technique, osteotomy was performed, as described previously [9, 21].

Based on the postoperative radiograph of patients suffering from mTFA overcorrection, a posteriori correction was simulated by correcting the mTFA to the preoperatively desired WBL ratio. The proportion of excessive osteotomy gap opening was subtracted from the preoperatively planned gap size (Miniaci) and compared to planning with JLCA correction. Additionally, the postoperative gap size was corrected to the size planned using the Miniaci method and additional JLCA correction. The resulting MPTA, WBL ratio, and JLCA were measured and compared to the planning with the JLCA correction.

A small clinical case series (*n* = 8) with a preoperative JLCA > 3 was conducted using preoperative planning with the additional JLCA correction described above. Postoperative radiographs were taken at least eight weeks following surgery and with full weight bearing. Radiographic measurements were made as described above.

### Statistical analysis

Data are presented as means and standard deviations (SD). The calculation was based on two groups: (1) Miniaci planning and (2) Miniaci planning with previous JLCA correction. Differences between the groups were calculated with ANOVA and the Kruskal–Wallis test for non-parametric parameters. Categorical parameters were compared using the chi-squared test, and Fisher’s exact test was used for categorical parameters in the case of small subgroups (*n* < 5). Person’s correlation analysis and multiple linear correlations were performed using GraphPad Prism 8 (San Diego, CA, US). A *p* value < 0.05 was considered significant. A post hoc test for the multiple logistic regression analysis was performed using G-Power (version 3.1, Institut für Experimentelle Psychologie, Heinrich Heine Universität, Düsseldorf, FRG) and an α-error of 5%. The sample size of *n* = 78 revealed a statistical power of 0.9 to identify a significant factor associated with malcorrection. Radiographic measurements were independently analysed by a senior orthopaedic fellow and orthopaedic consultant with an interval between repeated analysis of 4 weeks. Intra- and interobserver reliability was assessed by calculating the intraclass correlation coefficient (ICC). Interobserver reliability ranged from 0.84 to 0.97 and intraobserver reliability ranged from 0.85 to 0.98, indicating high reliability, as reported previously [11, 39]. High intra- and interobserver reliability were measured for the planned osteotomy gap (0.99/0.98) and without JLCA correction (0.95/0.97).

## Results

### Patient demographics

Based on 120 screened cases, 78 full-leg radiographs that met the inclusion criteria and had accurate radiographs available were included. The demographic data of the study cohort are summarised in Table 1. Long postoperative leg standing radiographs were taken 2–3 months postoperatively.

**Table 1:** Patient demographics

Parameters	Total (*n* = 78)
Age (years)^a^	46.0 ± 9.6
Sex (male/female)	54/24
BMI (kg/m^2^)^a^	26.8 ± 3.2
Flow-up (in days)^a^	68.6 ± 51.0
Medial OW HTO	78 (100.0%)

*OW HTO* open-wedge high tibial osteotomy

^a^mean ± SD

### Radiological measurements and correction error

Data from the radiographic analysis of pre-and postoperative radiographs are shown in Table 2. Averaged values demonstrate a divergence of the preoperatively planned and postoperatively achieved WBL ratio of 3.5% without being significantly different from each other (*p* > 0.05). Considering a divergence of ± 5% in each case as miscorrection revealed 32.1% cases with an overcorrection and 5.1% undercorrection for the preoperatively aimed WBL ratio.

**Table 2:** Radiological measurements and correction errors

Parameters (in °)	Preoperative	Postoperative	Difference
HKA^a^	6.5 ± 10.8 (varus)	1.9 ± 1.5 (valgus)	8.5 ± 12.3
MAD (in mm)	21.2 ± 11.3	6.9 ± 5.1	28.1 ± 16.4
MPTA^a^	85.4 ± 1.9	91.2 ± 2.8	5.8 ± 0.8
LDFA^a^	88.6 ± 1.6	88.0 ± 1.9	0.6 ± 0.3
JLCA^a^	2.5 ± 1.8	2.0 ± 1.4	0.5 ± 0.4
JLO^a^	2.1 ± 1.7	2.5 ± 1.9	0.5 ± 0.3
WBL ratio^a^	54.2 ± 3.7*	56.6 ± 9.7	3.5 ± 6.2
Correction error			
Overcorrection > 5% aimed correction		32.1%	
Undercorrection < 5% aimed correction		5.1%	

*n* = 78

*planned using Miniaci method; WBL weight-bearing line ratio in % of the tibial plateau width (medial 0%, lateral 100%)

^a^mean ± SD

### Factors affecting postoperative overcorrection

Pearson correlation (Table 3) analysis revealed preoperative BMI (*p* < 0.04), JLCA (*p* < 0.0001) and the divergence of the osteotomy level (*p* = 0.0005) as significant factors influencing the rate of postoperative overcorrection. In a multiple linear regression analysis (Table 4), only the preoperative JLCA and divergence of the osteotomy level were identified as two critical variables associated with the rate of postoperative overcorrection. Neither one explained the distribution of the postoperative WBL ratio, indicating that it was influenced by several factors.

**Table 3:** Pearson correlation analysis of factors affecting the rate of overcorrection

Parameters	Coefficient of correlation	*p* value*
Age	0.100	0.4108
Weight (BMI)	0.224	**0.0443**
HKA	0.064	0.5961
Preoperative JLCA	0.416	**0.0001**
MPTA	0.035	0.7556
LDFA	0.176	0.1331
JLO	− 0.122	0.3004
Osteotomy level	0.380	**0.0005**

*n* = 78; Person correlation test was performed to determine the associated preoperative factors that affect the rate of overcorrection

*A *p* value of < 0.05 indicates statistical significance (bold)

**Table 4:** Multiple linear regression analysis of factors associated with the rate of overcorrection (*R* = 0.494, *R*^2^ = 0.244, *R*^2^_adj_ = 0.219, *p* < 0.001)

Dependent variable	Explicative variable(s)	Unstandardised coefficients		Standardised coefficients	
		B	SE (B)	β	*p* value*
Postoperative intersection of the mTFa with the tibia plateau (in %)	Preoperative JLCA	1.519	0.504	0.348	**0.004**
	Osteotomy level	0.207	0.086	0.277	**0.020**

*n* = 78; Multiple linear correlation was performed to determine the associated preoperative factors that affect the rate of overcorrection

*A *p* value of < 0.05 indicates statistical significance (bold)

Based on the factors influencing the rate of postoperative overcorrection and the definition above of over and undercorrection, the data were categorised into three scales of preoperative JLCA. As illustrated in Table 5, 62.5% of the cases with postoperative overcorrection clustered in the group with preoperative JLCA ≥ 4°. A Chi-square test of this distribution confirmed a significant association (*p* < 0.05) between preoperative JLCA and the rate of postoperative malcorrection. Intergroup comparison of each JLCA scale using ANOVA analysis revealed statistically significant differences in all groups for the postoperative WBL ratio (*p* < 0.01).

**Table 5:** Preoperative JLCA effects on the rate of preoperatively aimed mTFA correction

Preoperative JLCA	Undercorrection	Optimal	Overcorrection
< 2	7.4% (2/27)	74.0% (20/27)	18.5% (5/27)
2–< 4	7.4% (2/27)	77.8% (21/27)	14.8% (4/27)
≥ 4	0% (0/24)	37.5% (9/24)	62.5% (15/24)

### Preoperative planning with and w/o JLCA correction and a posteriori validation

Preoperative JLCA correction almost did not influence the osteotomy gap size and subsequent MPTA in patients with a preoperative JLCA < 4. This analysis did not include patients with a preoperative JLCA ≤ 2 since no modified planning was applied according to the JLCA-2/2 formula. Given the low rate of over-corrections in the group with preoperative JLCA > 2°–< 4°, an a posteriori correction was not considered in this group. In cases with a preoperative JLCA ≥ 4°, a JLCA correction resulted in a distinct reduction of the preoperatively planned osteotomy gap by an average of 1.4 mm and significantly (*p* < 0.05) reduced MPTA in the osteotomy simulation (Table 6). If the amount of overcorrection corrected the osteotomy gap planned by the Miniaci method, the gap size was almost identical to the one planned with the additional JLCA correction. In addition, the resulting MPTA was reduced in comparison to the preoperatively planned MPTA (*p* < 0.05) and postoperatively achieved MPTA (ns). No differences in JLCA correction in preoperative planning were detected for LDFA.

**Table 6:**
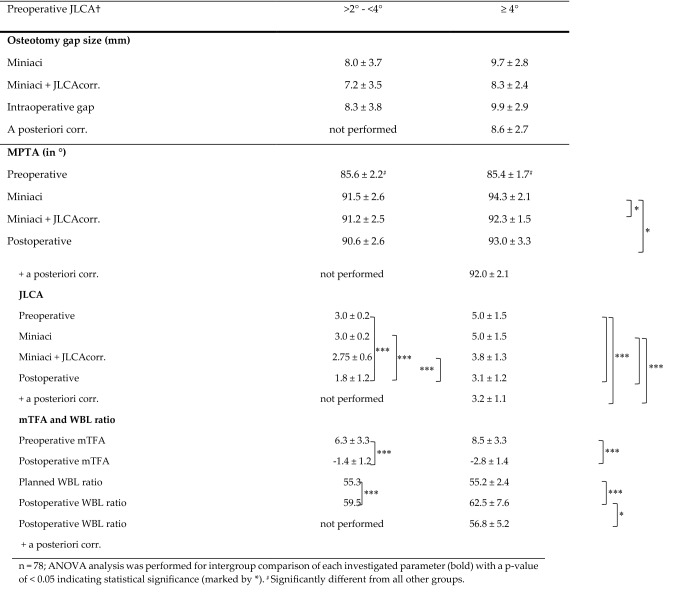
Preoperative planning with and w/o JLCA correction and a posteriori validation

### Case series with preoperative planning, including JLCA correction

**Fig. 3:**
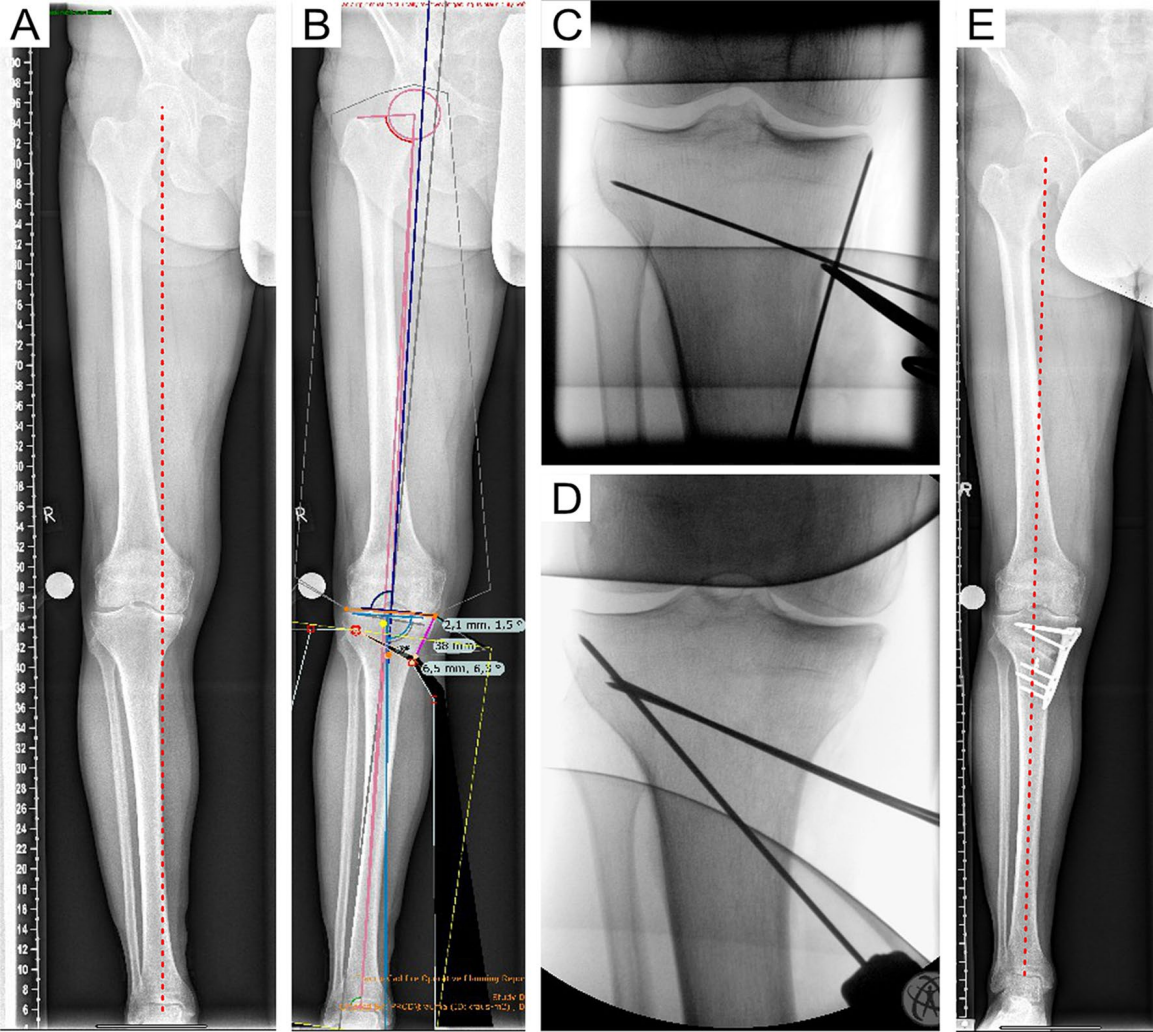
Representative case of a 54-year-old patient with medial compartment OA and 6° varus deformity. Based on a long-leg weight-bearing X-ray (**A**), a deformity analysis was conducted preoperatively. The osteotomy was simulated (TraumaCAD™) based on the technique described by Miniaci but with additional JLCA correction using the JLCA-2/2 formula due to a preoperative JLCA of 4°. The preoperative simulated osteotomy level localised at the medial cortex of the tibia was precisely realised during surgery (**C**). Subsequent OW HTO was secured by a hinge-protection k-wire (**D**). Postoperatively (**E**), the mTFA intersected the tibial plateau at 52%, resulting in a 3° divergence compared to the preoperatively planned intersection point (55% tibial width)

Eight patients (41.1 ± 4.2 years) with preoperative varus of 6.6 ± 2.5° and 4 ± 0.5° JLCA were operated by OW HTO using the preoperative planning (MPTA 93.3 ± 1.5°; JLCA 3.7 ± 0.6°; LDFA 89.7 ± 1.2°, osteotomy gap 9.2 ± 0.3 mm) with additional JLCA correction (MPTA 89.0 ± 1.1°; JLCA 3.0 ± 0.2°; LDFA 89.3 ± 1.1°, osteotomy gap 8.0 ± 0.7 mm) and exact translation of the osteotomy level intraoperatively (Fig. 3). Postoperative analysis (MPTA 90.3 ± 1.5°; JLCA 2.6 ± 0.6°; LDFA 90.6 ± 2.5°, mTFA–1.4 ± 1°) revealed a divergence of the mTFA of 3.4 ± 1.9% (range 0.3–5%).

## Discussion

The primary finding of this study was that preoperative JLCA and a divergent osteotomy level were identified as risk factors for postoperative overcorrection in varus malalignment correction. Preoperative JLCA correction can be simulated within preoperative planning and contributes to a more precise correction of the mechanical weight-bearing axis and a reduced postoperative MPTA.

The clinical relevance of overcorrection was shown in a study by Hohloch et al., who demonstrated in a comparative study of HTOs aiming for postoperative 50–55%, 55–60% and > 60% mTFA intersection significantly better results in terms of Lysholm Score and Knee Injury and Osteoarthritis Outcome Score (KOOS) for a 50–55% intersection [13]. This issue has been controversial, and additional preoperative assessments may be necessary to anticipate the clinical outcome [36]. However, in the authors’ opinion, an accuracy of ± 1.5°, as mentioned before, seems necessary when performing varus correction osteotomies because biomechanical studies have observed a significant load shift beginning at 3° of varus [4, 8]. Therefore, a ± 5% WBL ratio divergence was selected for this study, corresponding to a ± 1.5° mTFA divergence. Based on this definition, we recorded 32% of over-corrections, which emphasised the problem of poor accuracy, although the prerequisites mentioned in the introduction were entirely realised. Similarly, in a comparative study including navigation-guided osteotomy correction, Schroeter et al. failed to achieve an accuracy of ± 1.5° divergence of the preoperatively planned and postoperatively achieved mTFA correction [37]. These findings point out a significant limitation of osteotomies to date, and further literature reports about a rate of malcorrection of 30–40%, which critically depends on the definition of malcorrection [32, 33, 35, 37, 38]. Studies reporting a low rate of malcorrection often defined a much wider corridor concerning the postoperative WBL ratio that was considered satisfying [20].

Regarding this dilemma, numerous studies have evaluated the factors influencing accuracy in varus osteotomy correction [33, 37, 38]. Our study identified BMI, preoperative degree of JLCA, and divergence in the osteotomy level as influencing factors. None of these factors explained the rate of overcorrection, and BMI did not withstand the multiple regression analysis, consistent with previous studies [12, 33, 34]. Regarding lateral tibial alignment, displaced hinge fractures were excluded in our study, as this can seriously affect the accuracy of valgus osteotomy [18]. Lee et al. reported an increased medial opening gap of 12 mm as a risk factor for lateral hinge fractures [22]. However, non-displaced hinge fractures cannot be ruled out entirely because no postoperative MRI or CT scans were performed routinely. Lateral hinge fractures can affect the maintenance of the osteotomy gap and lead to correction loss, resulting in undercorrection [19, 26].

To the authors’ best knowledge, the importance of a divagating osteotomy height at the medial tibia border has not been identified before as a risk factor for overcorrection. Minimised divergence from preoperative planning can be realised by precisely translating the preoperatively planned osteotomy level during the surgery. The importance of osteotomy inclination and positioning of the hinge in three-dimensional thinking has recently been clarified for the sagittal plane [5, 28]. The posterior tibial slope tends to increase when the osteotomy inclination angle in the sagittal plane is inclined posteriorly with respect to the medial tibial plateau. Such a reverse effect of the osteotomy inclination angle may be apparent in the coronal plane, causing a divergence of the planned WBL ratio [29]. However, no comprehensive study has examined the effect of the osteotomy inclination in the frontal plane and its correlation with the osteotomy gap size and change of the mechanical axis in the valgus osteotomy. Some investigators have already emphasised the importance of 3D planning and its clinical realisation using patient-specific cutting devices [6, 30]. Whether this development is superior to the standard technique for osteotomy remains debated. Nevertheless, the inclination of the osteotomy plane and hinge positioning should be focused on further research and considered when considering the accuracy of the osteotomy.

The most common contributor to poor accuracy in varus correction osteotomy is the amount of intraarticular deformity that cannot be included in the bony correction and that can be estimated only by the JLCA measurement. As in this study, previous work has identified increased preoperative JLCA as a risk factor for postoperative overcorrection [33, 40]. Significant effort was made to mathematically estimate the change in the JLCA from pre to postoperatively, between supine and weight-bearing radiographs, and with and w/o stress radiographs [7, 25, 35]. JLCA change from the pre- to the postoperative conditions becomes even more challenging since it is clinically established to perform a medial release to unload the medial compartment effectively [2]. Therefore, to date, there is no final consensus on how to introduce JLCA correction in preoperative planning.

Given these difficulties, Micicoi et al. proposed a simple equation “JLCA-2/2” to estimate the intraarticular varus proportion and preoperatively correct this amount by performing a virtual intraarticular osteotomy [25]. Our study provides the first evidence that this additional planning step improves the accuracy of varus correction osteotomies and yields reduced postoperative MPTA values. Because no stress radiographs were taken in this study, we could not compare the correction amount calculated by the JLCA-2/2 formula to related equations proposed by stress or supine radiographs [7]. However, this study proves that a specific JLCA correction is necessary when shifting the mTFA, thereby reducing the force momentum that causes varus gaping in the lateral compartment. Care should be taken when performing an intraoperative JLCA correction using stress radiographs, as described by others [20] because the intraoperative alignment technique using a rod or cautery cable is inaccurate [41].

Some critical limitations should temper this study’s conclusions. The study was conducted retrospectively with a heterogeneous cohort of patients. However, narrow inclusion criteria were applied to eliminate numerous factors affecting osteotomy accuracy, the reason for our study's high number of excluded patients. Depending on the size of the osteotomy gap, the medial release might have been performed more or less radical and with or w/o refixation. Final osteotomy gap fixation can sometimes be changed slightly by rounding up or down and integrating bone loss depending on the thickness of the sawing blade. Additionally, there might be a small postoperative change in the mTFA over the long course. However, in our study, postoperative radiographs were taken after 2–3 months, rendering our findings early radiographic results. However, current internal plate fixateurs allow the lateral alignment of the proximal epiphysis of the tibia to be maintained, minimising the secondary loss of correction [24]. The findings based on the a posteriori correction and small case series must be validated by a more extensive clinical study and interpreted cautiously.

## Conclusion

Based on previously reported requirements for accurate osteotomy correction in varus knees, higher accuracy may be achieved by preoperative JLCA correction using the JLCA-2/2 equation and precise conversion of the preoperatively planned osteotomy level into surgery. When treating patients with an elevated BMI, caution should be taken to avoid overcorrection.

## Abbreviations

BMI: Body mass index
CORA: Center of rotation and angulation
CW: Closed wedge
DFO: Distal femur osteotomy
HTO: High tibial osteotomy
HKA: Hip knee angle
JLCA: Joint line convergence angle
JLO: Oblique joint line
LDFA: Lateral distal femur angle
MAD: Mechanical axis deviation
mTFA: Mechanical tibio-femoral axis
MPTA: Medial proximal tibia angle
OA: Osteoarthritis
OW: Open wedge
WBL: Weight-bearing line
